# Shell-ferromagnetism of nano-Heuslers generated by segregation under magnetic field

**DOI:** 10.1038/srep28931

**Published:** 2016-07-14

**Authors:** A. Çakır, M. Acet, M. Farle

**Affiliations:** 1 Muğla University, Department of Metallurgical and Materials Engineering, 48000 Muğla, Turkey; 2 Faculty of Physics, Duisburg-Essen University, D-47057 Duisburg, Germany

## Abstract

We report on a new functional property in an AF martensitic Heusler Ni_50_Mn_45_In_5_, which when annealed at high temperatures under a magnetic field, segregates and forms Ni_50_Mn_25_In_25_ Heusler precipitates embedded in a Ni_50_Mn_50_ matrix. The precipitates are paramagnetic whereas the matrix is antiferromagnetic. However, the spins at the interface with the Ni_50_Mn_50_ matrix align with the field during their nucleation and growth and become strongly pinned in the direction of the applied field during annealing, whereas the core spins become paramagnetic. This shell-ferromagnetism persists up to 600 K and is so strongly pinned that the remanent magnetization remains unchanged, even when the field is reversed or when the temperature is cycled between low temperatures and close to the annealing temperature.

The martensitic transition in Ni-Mn-based Heusler materials is the source of their functionality related to the prominent magnetic shape memory, giant magnetoresistance, baro and magnetocaloric, and exchange bias effects^1^. The stoichiometry of the materials showing these effects lies in a narrow compositional range where a martensitic transition takes place from a high-temperature cubic austenite state with long-range ferromagnetic (FM) ordering to a low temperature modulated tetragonal martensite state, where the ordering is usually short-range frustrated antiferromagnetic (AF)^2^. Martensitic Heuslers with higher Mn concentrations are antiferromagnetic (AF) and therefore, are thought to be of less functional relevance^3^. However, the more recent observation of zero-field-cooled exchange bias^4^,^5^, occurring precisely at high Mn concentrations and low temperatures, added a new aspect to the wide palette of physical phenomena in martensitic Heuslers and demonstrated that such materials can gain further functionality through the presence of strong AF coupling. To which new effects and functionalities strong AF coupling can lead to is a relatively unexplored area. Here, we report on a new functional property in an AF martensitic Heusler Ni_50_Mn_45_In_5_. When annealed at high temperatures under a magnetic field, the compound segregates and forms Ni_50_Mn_25_In_5_ Heusler precipitates embedded in a Ni_50_Mn_50_ (NiMn) matrix. The precipitates are paramagnetic whereas the matrix is antiferromagnetic. However, the spins at the interface with the NiMn matrix, which align with the field during their nucleation and growth, become strongly pinned in the direction of the applied field during annealing whereas the core spins become paramagnetic. This shell-ferromagnetism persisting up to ~600 K is so strongly pinned that the remanent magnetization remains unchanged, even when the field is reversed. Such field-proof permanent memory, can be interesting for applications at reduced dimensions and high temperatures.

## Results

**Figure 1:**
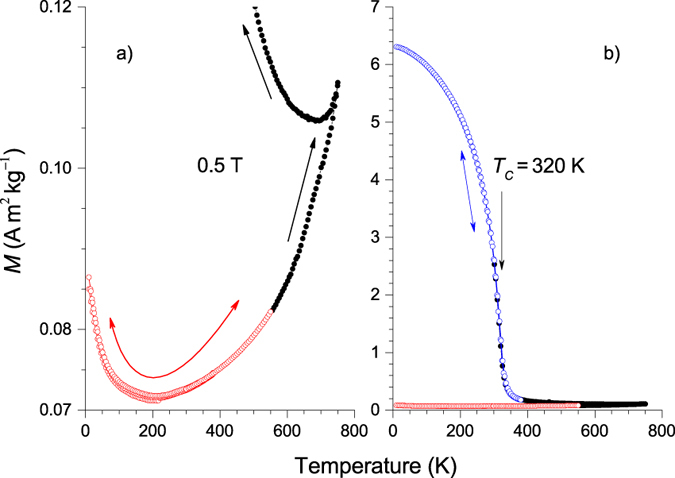
Segregation of Ni_49.6_Mn_45.5_In_4.9_ into ferromagnetic and antiferromagnetic components observed in the temperature dependence of the magnetization in two vertical scalings. (**a**) Low-scaled vertical axis. The data shown by open red symbols are retraceable in temperature as indicated by the red double-headed arrow. The data shown with black filled symbols are not retraceable as indicated by the black arrows. (**b**) The vertical axis on a larger scale with the data (open blue symbols) showing ferromagnetic behavior. The data is retraceable as indicated by the double-headed blue arrow. The red and black data are the same as those in part (**a**).

The nature of AF ordering in Ni-Mn based functional martensitic Heuslers in the form Ni_50_Mn_25−*x*_*X*_*x*_ (*X*: Ga, In, Sn, Sb) is little known. Although the predominant interaction in the Mn-rich case (approximately *x* ≤ 10) is AF, a Néel temperature has never been reported for any of these compounds. To look closer into the details of the AF ordering in Mn-rich martensitic Heuslers, we began by investigating the structural and magnetic properties of a Ni_49.6_Mn_45.5_In_4.9_ sample. Earlier investigations have shown that this alloy has a tetragonal L1_0_ martensite structure and is essentially AF at room temperature^6^. It exhibits mixed FM-AF interactions at low temperatures. In an attempt to locate the Néel temperature *T*_*N*_, we measured the temperature dependence of the magnetization *M*(*T*) in a field of 0.5 T in the temperature range 10 ≤ *T* ≤ 750 K. Instead of observing a cusp-like feature characteristic of a *T*_*N*_-point, we find as seen in Fig. 1a that *M*(*T*) runs through a minimum at around 200 K and then continues to increase with increasing temperature. *M*(*T*) is found to be reversible in the range 10 ≤ *T* ≤ 550 K (red data) as shown with the double-headed red arrow. If the direction of temperature-change is reversed at *T* > 550 K, *M*(*T*) (black data) becomes irreversible as shown by the black arrows. The irreversibility is shown here for the case when the sample is cooled from 750 K. When the temperature is decreased, *M*(*T*) does not follow its initial path and begins to increase with decreasing temperature. With the vertical scale plotted in a broader range in Fig. 1b, *M*(*T*) is seen to exhibit FM behavior (blue data) with a Curie temperature *T*_*C*_ ≈ 320 K. *M*(*T*) is again reversible in the range 10 ≤ *T* ≤ 550 K as indicated with the double-headed blue arrow. These features indicate that warming the sample to *T* > 550 K leads to a phase separation with at least one of the phases having a larger magnetization than the remaining part of the alloy. The size of *T*_*C*_ suggests that the large-magnetization component is around the Ni_2_MnIn stoichiometry.

**Figure 2:**
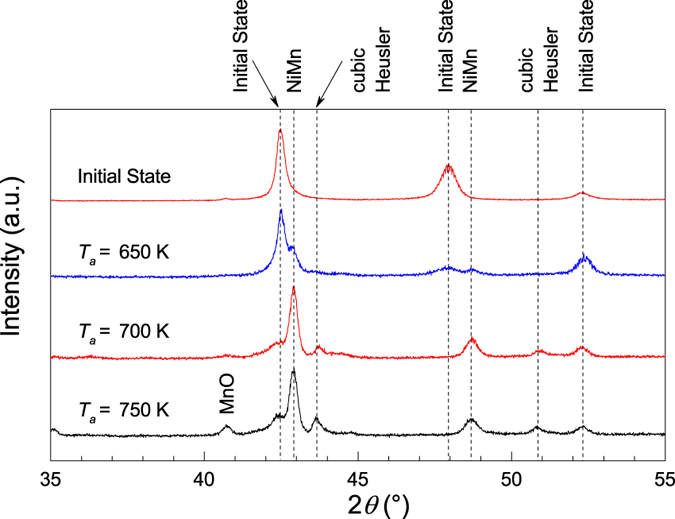
X-ray diffraction at room temperature of the initial state and after decomposition at 650, 700, and 750 K. The intensity of the peaks of the initial state weaken while those of NiMn and the cubic Heusler strengthen as *T*_*a*_ increases. A peak related to MnO is also observed for *T*_*a*_ = 750 K.

To identify the phases in the sample and their evolution, we annealed the samples under a magnetic field of 1 T for 24 h at the annealing temperatures *T*_*a*_ = 650, 700 and 750 K in an oven installed on a magnetometer. Subsequently, x-ray diffraction (XRD) experiments were carried out. The XRD results along with the data for the initial state of the sample are shown in Fig. 2. The initial state of the sample corresponds to the non-modulated tetragonal L1_0_ martensite structure as had also earlier been identified^6^. The data corresponding to the 750 K phase-separated state shows that the L1_0_ martensite structure decomposes into different crystallographic phases. According to Rietveld refinements, the components correspond to the initial state, to austenite Heusler, and to tetragonal NiMn as indicated in Fig. 2. A small amount of MnO is also encountered especially in the *T*_*a*_ = 750 K-data. The vertical dashed lines guide the evolution of the peak positions and intensities with respect to increasing *T*_*a*_. The peaks related to the initial state lose intensity, while those corresponding to NiMn and the cubic Heusler gain intensity.

Although the lattice parameters of the initial state and the L1_0_ NiMn state are in agreement with their respective values corresponding to those of the bulk, the cubic cell parameter of the austenite Heusler is estimated to be about *a* = 5.88 Å which is considerably smaller than that of Ni_2_MnIn for which *a* ≈ 6.07 Å^6^. This difference should be due to the small size of the precipitate and/or strong strains arising from the lattice mismatch of the tetragonal and cubic phases. Nevertheless, it is a fact that the precipitates are FM below *T*_*C*_ = 320 K being very close to that of the Ni_50_Mn_25_In_25_ Heusler with 2-1-1 stoichiometry.

The phase separation at 750 K is not complete after 24 h, and some initial phase remains intact. But, the analysis of the x-ray spectra show that the main tendency is towards the stabilization of the components Ni_50_Mn_25_In_25_ and Ni_50_Mn_50_. This follows from the the decomposition process


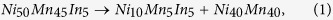


of which the right side can be considered as


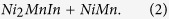


If fully decomposed into these components, the material would be made up of 24% Ni_2_MnIn and 76% NiMn. Assuming the contribution to the measured magnetization from AF NiMn to be zero at low temperatures, the total magnetization is then entirely due to FM Ni_2_MnIn. Since the magnetization of Ni_2_MnIn is 75 Am^2^kg^−1^ 6, the maximum attainable magnetization in the fully phase-separated state of Ni_50_Mn_45_In_5_ is 24% of this amount, namely 18 Am^2^ kg^−1^. The measured magnetization of the 750 K-annealed sample at 5 K is 6.2 Am^2^ kg^−1^ indicating that about one-third of the sample has decomposed within the 50-minute time-window of the experiment between 650 and 750 K at which decomposition is significant.

**Figure 3:**
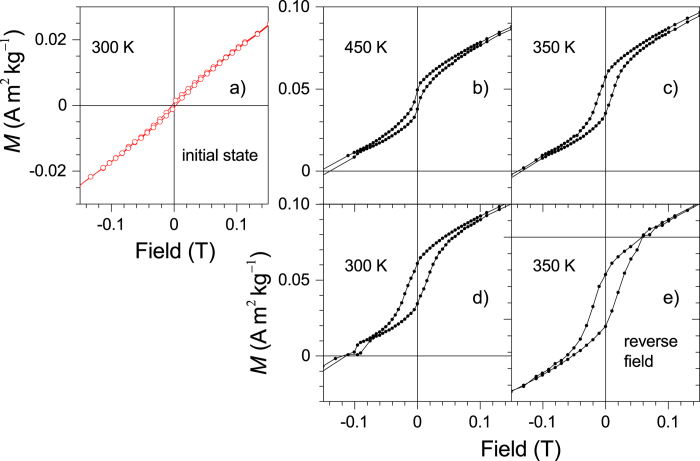
The field dependence of the magnetization shown in the range −0.15 ≤ *μ*_0_*H* ≤ 0.15 T. (**a**) The initial state at 300 K. (**b**) After segregation at 650 K measured at 450 K (**c**) at 350 K, and (**d**) at 300 K. (**e**) Measurement at 350 K of a sample segregated in a reverse field showing the (reverse) downward shift of the loop.

High temperature *M*(*H*) measurements (*T* ≥ 300 K) made in the field-range −4.5 ≤ *μ*_0_*H* ≤ 4.5 T are shown in Fig. 3 in the range −0.15 ≤ *μ*_0_*H* ≤ 0.15 T. In the initial state at 300 K (Fig. 3a), the system is predominantly AF. The sample was then taken to *T*_*a*_ = 650 K where it remained for about 1 h in 4.5 T and partially decomposed. Subsequently, the temperature was lowered to 450 K and a magnetization loop was measured (Fig. 3b). The loop is shifted vertically. The hysteresis in the loop broadens and the vertical shift becomes more apparent when the sample is further cooled to 350 K and then to 300 K (Fig. 3c,d). The shift and the form of the loop remain the same whether the sample is cooled to the measurement temperature in a field or in the absence of a field and also when the field is cycled backwards (not shown here). The shift is positive if the applied field during segregation is positive and negative if the field is negative. The negative shift is shown in Fig. 3e for a sample that was annealed at 650 K in a reverse field (−4.5 T) and measured at 350 K. In all cases, a remanence in the magnetization is observed with the sign depending on the direction of the applied field during segregation. Furthermore, the remanence is restored to its initial value after the field is cycled through negative values. The shift prevails also when the temperature is cycled to low temperatures and again back to high temperatures. If the sample is annealed in zero-field, there is no shift. By comparing these magnetizations to the saturation magnetization of Ni_50_Mn_25_In_25_ we estimate that 0.01% of the sample decomposes to give rise to these effects when annealed at 650 K for 1 h under 4.5 T. These data are also presented in the range −2 ≤ *μ*_0_*H* ≤ 2 T in Supplementary materials to provide a comparative impression on the size of the effect.

## Discussion

The cause of a vertical shift of a hysteresis loop is related to strong pinning of the spin alignment in a direction which is determined by the applied external field while precipitation initiates and progresses. Ni_50_Mn_25_In_25_ is cubic and PM at these high temperatures, and NiMn is tetragonal and AF. Any prevailing initial phase is also AF. As precipitation progresses in a magnetic field, the spins at the interface between the growing Ni_50_Mn_25_In_25_ components and the surrounding AF NiMn initial phase matrix are given a preferred orientation, essentially along the field direction. The surrounding AF matrix then takes over to pin the interface spins in the field direction giving rise to the remanent alignment of the interface spins when the field is removed.

**Figure 4:**
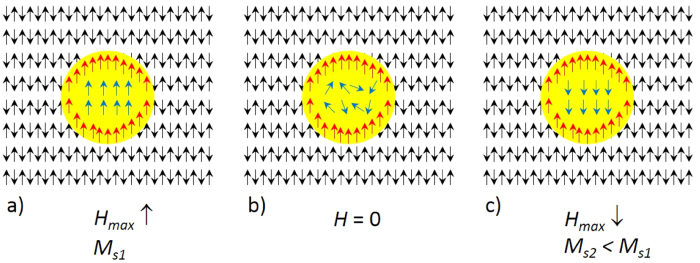
A model description of the resulting magnetic configuration after the sample decomposes in a magnetic field. The yellow area represents the Heusler and the surrounding antiparallel aligned spins represent Ni-Mn. The red arrows depict the spins aligned in the applied field direction acquired during segregation and are strongly pinned by the interlayer exchange. The blue arrows depict those spins that only align in the presence of an external field. (**a**) All spins are aligned parallel to the external field (in this case also the initial maximum field of the measurement). (**b**) The external field is zero. Core spins are randomly aligned but interlayer spins remain aligned. (**c**) Core spins align opposite in the reverse field direction. Interface spins remain aligned in the direction of the field applied during segregation. The resulting magnetization is smaller than in (**a**) giving rise to a vertical shift in the hysteresis.

The sequence of events leading to the vertically shifted loop is illustrated in Fig. 4. The diagram is schematic and is meant only to describe the main features of the data. The yellow area represents the precipitate surrounded by the AF matrix. The red arrows indicate ferromagnetically aligned interfacial spins which are strongly pinned by the anisotropy of the AF proximity. These spins are most likely to align non-collinearly with a complex configuration but at the same time have a net magnetization along the segregating field direction. Since we do not know the exact configuration, we show them here as parallel aligned for simplicity. The blue arrows represent the ‘loose’ spins within the paramagnet that can align only when a field *H* is applied as in Fig. 4a. In this case *H* = *H*_*max*_, and the saturation magnetization is *M*_*S*1_. When the field is removed, the inner spins disorder while the interfacial spins remain pinned in their original direction, which was given by the field during precipitation as shown in Fig. 4b. When the field is reversed to −*H*_*max*_, the internal spins rotate in the direction of the field while the spins at the interface remain essentially in their original direction so that the final saturation magnetization *M*_*S*2_ is smaller than *M*_*S*1_ (Fig. 4c). The strong uniaxial anisotropy of the interfacial spins leads to the vertically shifted hysteresis loops – the direction of the shift being determined by the direction of the field during precipitation.

Although this picture can describe the vertical shift, it cannot account for the observed hysteresis in *M*(*H*) since a most likely gradual decrease in the strength of the pinning from the interface to the core region is not taken into account. Neither is the effect of strain, due to the lattice mismatch between the L1_0_ AF matrix and the cubic Heusler precipitate, and its coupling to the magnetic structure is considered. In particular, the effect of mechanical strain along with any enhancement in the spin-orbit coupling, which are not taken into account here, can be major sources for the hysteresis.

Our results exhibit that non-stoichiometric Ni-Mn-based Mn-rich Heusler compounds are metastable and tend to decompose predominantly into the L2_1_ 2-1-1 Heusler stoichiometry and L1_0_ NiMn. When decomposing under a magnetic field, the magnetic field interacts with the spins of the growing precipitates during their nucleation and growth. If the size of the X_2_YZ Heusler precipitates are sufficiently small, interesting magnetic configurations can occur. Here, we obtain a FM configuration in the interface although the core is paramagnetic. As the precipitate grows, so does the interface. Even though it is presently not possible to give a size for the precipitates, it can be argued that if the size is small, the interface-to-volume ratio is large so that the effect at the interface on the features of the magnetization from the surface (pinned spins) is comparable to those of the volume (loose spins), giving rise to vertically asymmetric magnetization loops. If the precipitate becomes too large the asymmetry feature is practically masked. Almost no vertical shift is observed in samples annealed at 750 K for 1 h under 5 T (see Supplementary material) and vanishes completely when annealed for longer periods at this temperature.

Vertical loop shifts of lower magnitude have been reported in FM/AF interfacial systems at low temperatures and around room temperature^7^,^8^,^9^,^10^,^11^, but never at high temperatures in paramagnetic regimes where the active magnetic configuration is constructed during the nucleation and growth of precipitates under a magnetic field. A thorough review on the magnetic proximity effect has also recently been provided^12^. The observation here of a vertical shift at high temperatures and of such high magnitude in a PM/AF interfacial system is attributed to the magnetic proximity effect. This arises because an interfacial FM layer occurs between a core paramagnet with strong FM correlations and an AF surrounding. The generation of this strong PM/AF proximity effect using a high-temperature segregation process in an external field brings a new aspect to interfacial magnetic effects.

In future works, by determining the size and controlling the growth of the segregating PM components, it should be possible to control the strength of the interlayer exchange coupling in practically a plethora of materials that segregate into components with different magnetic coupling. The consequences of this strong magnetic proximity effect can motivate further research on this alternative field-proof permanent memory and on the understanding of interfacial magnetic coupling and the mechanism of the occurrence and growth of shell-ferromagnetism in precipitates.

## Methods

The samples were prepared by arc melting followed by annealing under 300 mbar Ar in sealed quartz ampules at 1073 K for 5 days. The samples were then quenched in water at room temperature. The composition of the sample was determined by EDX analysis on a scanning electron microscope. The magnetization was measured in the range 5 ≤ *T* ≤ 750 K using a squid magnetometer. X-ray diffraction was carried out using Cu K-alpha radiation.

## Additional Information

**How to cite this article**: Çakır, A. *et al.* Shell-ferromagnetism of nano-Heuslers generated by segregation under magnetic field. *Sci. Rep.* **6**, 28931; doi: 10.1038/srep28931 (2016).

## Supplementary Material

Supplementary Information
