# Supplementary material for: Shell-ferromagnetism of nano-Heuslers generated by segregation under magnetic field
Source: Sci Rep. 2016 Jul 14;6:28931. doi: 10.1038/srep28931 (PMC4944126; doi:10.1038/srep28931)
Supplement: Supplementary Information [file srep28931-s1.pdf]

Shell-ferromagnetism of nano-Heuslers generated by segregation under magnetic field

A. Çakır[1,*], M. Acet[2], and M. Farle[2]

[1]Muğla University, Department of Metallurgical and Materials Engineering, 48000 Muğla, Turkey
[2]Faculty of Physics, Duisburg-Essen University, D-47057 Duisburg, Germany
*cakir@mu.edu.tr

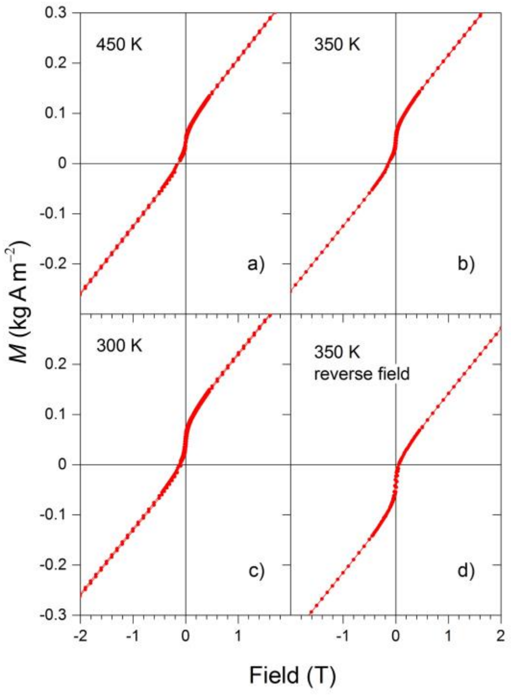

Fig. 1. The field dependence of the magnetization shown in the range $-2 \leq \mu_0 H \leq 2$ T. a) 450 K, b) 350 K, c) 300 K, d) 350 K and segregated in reverse field.

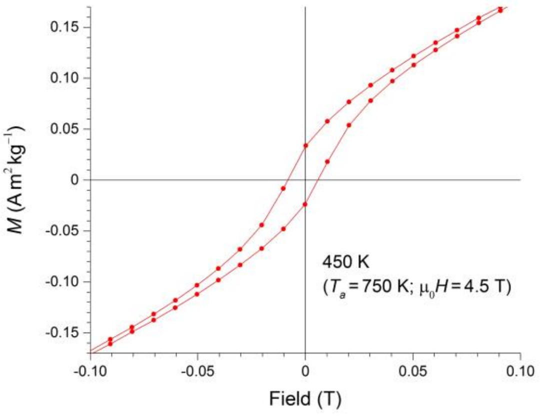

Fig. 2. *M(H)* at 450 K of a sample segregated at 750 K for 1 h under 5 T. The vertical shift is practically absent.
